# Advancing Discovery of Snail Mucins Function and Application

**DOI:** 10.3389/fbioe.2021.734023

**Published:** 2021-10-11

**Authors:** Maxwell McDermott, Antonio R. Cerullo, James Parziale, Eleonora Achrak, Sharmin Sultana, Jennifer Ferd, Safiyah Samad, William Deng, Adam B. Braunschweig, Mandë Holford

**Affiliations:** ^1^ Department of Chemistry and Biochemistry, Hunter College, New York, NY, United States; ^2^ Advanced Science Research Center, Graduate Center of New York, Graduate Department of Biochemistry, New York, NY, United States; ^3^ PhD Programs in Biochemistry and Chemistry Graduate Center of the City University of New York, New York, NY, United States; ^4^ PhD Program in Biology Graduate Center of the City University of New York, New York, NY, United States; ^5^ Department of Invertebrate Zoology, The American Museum of Natural History, New York, NY, United States

**Keywords:** mucins, mucus, snails, biopolymer, biotechnology, cosmetics

## Abstract

Mucins are a highly glycosylated protein family that are secreted by animals for adhesion, hydration, lubrication, and other functions. Despite their ubiquity, animal mucins are largely uncharacterized. Snails produce mucin proteins in their mucous for a wide array of biological functions, including microbial protection, adhesion and lubrication. Recently, snail mucins have also become a lucrative source of innovation with wide ranging applications across chemistry, biology, biotechnology, and biomedicine. Specifically, snail mucuses have been applied as skin care products, wound healing agents, surgical glues, and to combat gastric ulcers. Recent advances in integrated omics (genomic, transcriptomic, proteomic, glycomic) technologies have improved the characterization of gastropod mucins, increasing the generation of novel biomaterials. This perspective describes the current research on secreted snail mucus, highlighting the potential of this biopolymer, and also outlines a research strategy to fulfill the unmet need of examining the hierarchical structures that lead to the enormous biological and chemical diversity of snail mucus genes.

## Introduction

**FIGURE 1:**
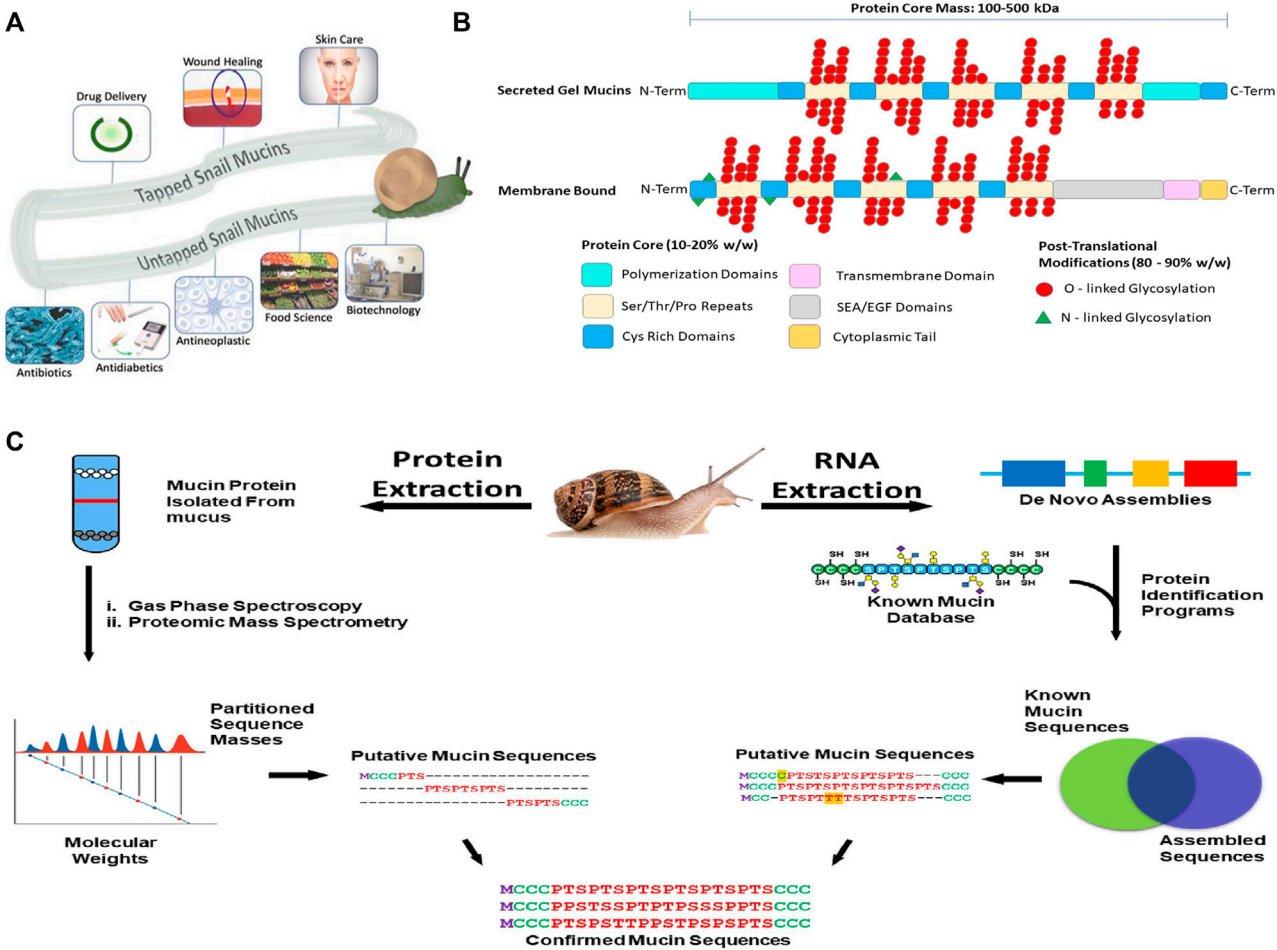
**(A)** Applications of snail mucus. Snail mucus has been used for skin care, wound healing and rejuvenation, and drug delivery. Snail mucus is being explored in food science, implant coatings, and other biotechnical sectors are currently researching mucins to be explored for potential use. **(B)** A 2-dimensional representation of the mucin structures. Mucins are characterized by two parts of their structure, their protein core, and their glycan branching. The protein core is a protein sequence of variable length depending on the mucin gene, which has been further modified with glycosylation branches. The Protein structure, however has multiple domains, and these domains vary depending on the function and the cellular location of the mucin. The glycan branches are sugar branches ranging from 3 to 18 sugars, and make up the majority of the mucin mass. Shown are 2 dimensional representations of the different types of mucins, and their stereotypical features. **(C)** Applying an integrated omics approach to identify snail mucin sequence, structure, and function. Path 1(left) extract crude mucin proteins and separate from the cellular debris to obtain sequence masses from spectroscopic and mass spectrometric analyses. Path 2(right) RNA extraction from mucus glands or whole animal followed by *de novo* assembly of mucin gene sequences to generate a database to BLAST against by a comparison of assembled sequences to a known mucin database, we obtain putative mucin sequences. Combining the proteomic and RNA pipelines we confirm the native type mucin sequence for further analysis.

Intrigue in the mucus slime trails left by snails and slugs date back to ancient Greece, where they utilized the mucus for its ability to reduce inflammation and the signs of aging (Ekin, 2018). Today snail mucus is still used in skin care products by various companies and is a growing market whose value is expected to approach $770 million by 2025 (Coherent Market Inisghts, 2018). Despite its commercial applications, the field of mucus research remains surprisingly underdeveloped. The primary constituent that is responsible for the properties of mucus are secreted mucins, a family of heavily glycosylated proteins produced in epithelial cells in most animals. Mucins are either bound to the plasma membrane or secreted out of the cell, and each type has major differences in their functions and capabilities (Dhanisha et al., 2018). Membrane-bound mucins are glycolipids that act as markers for cell signaling and also protect the cell from extracellular affronts that might lead to damage, such as infections and physical strain (Van Putten and Strijbis, 2017). Secreted mucins can be either gel forming or non-gel forming biopolymers. Secreted biopolymers form mucous membranes macroscopic scale. These mucosal membranes account for a large portion of the surface area of multicellular organisms exposed to the environment. In humans, mucosal membranes account for 99% of the bodies surface area (Sompayrac, 2012; Ma et al., 2018; Cerullo, 2020). Each snail species secretes multiple distinct functional mucuses. The mucus produced by a snail’s foot is used for adhesion and for lubrication, allowing the snail to stick onto or walk across any surface, even while inverted. Additionally, the mucus produced on the back of the snail is used for microbial defense and tissue hydration. Certain snail species have specialized uses for mucus. For example, *Falsilunatia eltanini* (Moon Snail) uses mucus to protect their eggs, and *Tikoconus costarricanus* (Costa Rican Land Snail), uses mucus for load-bearing activities, such as to hide from the Sun on the bottom of leaves during droughts (Gould et al., 2019; Barrientos, 2020). Recent advances in omics (genomic, transcriptomic, proteomic, glycomics) technologies have expanded the exploration of gastropod mucins as a scientific resource with wide ranging applications across chemistry, biology, biotechnology, and medicine. For example, the antimicrobial properties of snail mucus are being used to combat disorders seen in humans ranging from gastric ulcers, to post-surgical-related infections (Amah et al., 2019; Gentili et al., 2020). Mucins are also being coupled with approved therapeutics in order to potentiate the drug’s abilities to cure diseases, such as diabetes and ulcerative colitis (Gugu et al., 2020). Additionally, snail mucins are being investigated in a vast array of other biotechnical applications that exploit their surfactant-like properties (Petrou and Crouzier, 2018). Despite their potential, little is known about how the hierarchical mucin structures account for their diverse functional properties. There is an unmet need to examine the biological and chemical diversity of snail mucin genes to elucidate the guiding principles that determine the diverse properties associated with each gene. This perspective article will highlight current applications of secreted snail mucus that demonstrate the potential of this biopolymer as a resource for biotechnological and biomedical advancements. We will also describe an integrated omics strategy for investigating the biological and chemical diversity of snail mucus genes (Figure 1).

### Structural Variations of Mucins

Mucins contain several domains that contribute to their overall function (Figure 1). The structural variation allows for their extensive biological diversity and unique physical characteristics. A tandem repeat domain located in the center of the protein backbone, rich in serine, threonine, and proline, serves an as anchor for glycosylation. Mucin glycans are predominantly O-linked, but minor amounts of N-linked glycans can be present (Corfield, 2015). The length of the glycosylation domain and number of repeats differs between mucins and imparts different chemical characteristics. Secreted mucins have cysteine-rich regions on both ends of the tandem repeat domain that are used for stabilization, providing disulfide bridge points for both inter- and intramolecular bonding. Additionally, these regions serve both to provide additional structural diversification, and allow for multimerization of mucins and other sulfur-rich biomolecules (Perez-Vilar and Hill, 1999).

Typically, N-acetylgalactosamine (GalNAc) is attached to the protein core via O-glycosidic bonds between the monosaccharide and either Ser or Thr residues (GalNAc[α1]-Ser/Thr). This forms the TN Antigen, which is commonly found in humans to be upregulated in certain cancers (Guillen-Poza et al., 2020). From there, galactose is appended to the structure (Gal[β1-3]GalNAc[α1]-Ser/Thr), forming the mucin core 1 O-glycan. O-glycans vary in size, from 2 to 20 sugar residues, and in composition, as other sugars such as N-acetylglucosamine (GlcNAc) and fucose (Fuc) are appended sequentially (Brockhausen, 1999). Sialic acids and mannose are also found in trace amounts. Sialic acids in particular, have been to known to play a major role in the immune properties of mucins. Sialic acid mediates cell-to-cell interactions, along with being able to mask antigens from human macrophages (Yan et al., 2020). Further, sialic acids are the major binding points for lectins, a common protein family found in the innate immune system (Bornhöfft et al., 2018). Additionally, secreted mucins also exhibit C-mannosylation, where C1 of mannose bonds with the indole ring in tryptophan, allowing for greater variation of tertiary structure (Linden et al., 2008).

Subtle changes within the mucin structure, in particular the amino acid sequence and glycosylation, can correspond to vastly different biological function (Bansil and Turner, 2006). While these proteins are predominantly carbohydrates by weight, up to 90%, both protein and glycan structures provide overall functional characteristics to the mucin (Linden et al., 2008). Additionally, individual mucins can have multiple glycoforms in normal and diseased states, and different populations of a single species can exhibit distinct glycoforms (Benktander et al., 2019). This diversity allows for organisms to individualize each mucin for specific physiological and environmental conditions. Overall there is little known about the genotype-to-phenotype connection of mucin genes that leads to the various functional properties. Several human mucin genes have been identified and there are at least 21 validated mucin coding genes, each with different biological activities (Rose and Voynow, 2006). In contrast, while many putative snail mucin genes have been identified none been validated. However, the lack of robust characterization of the genetics and structural differences between snail mucuses has not precluded their application to address pressing medical and biotechnological materials needs.

### Snail Mucins as Antimicrobial Agents

Antibiotic-resistant bacteria are becoming an increasingly prevalent issue without many viable solutions. Because mollusks lack adaptive immunity, they depend on physical barriers and innate immunity for protection against pathogenic agents (Gerdol, 2017). For most snails, the foot has the most contact with surfaces that are contaminated with pathogens and parasites, and secretion of mucus along the feet protects against such microbes. One of the earliest mucuses evaluated for antimicrobial activity was that of *Achatina fulica* (Giant African Land Snail) (Table 1) (Iguchi et al., 1982). Mucus from *A. fulica* (Mumuni et al., 2020) demonstrated promising antibacterial activity against the Gram-positive bacteria, *Bacillus subtilis* and *Staphylococcus aureus*, and the Gram-negative bacteria, *Escherichia coli* and *Pseudomonas aeruginosa* (Table 2). The mucus secretions of *A. fulica* inhibited the bacterial growth of both *S. aureus* and *S. epidermidis* when applied via wound dressing films on a mouse model (Santana et al., 2012). The wound dressings improved the maturation of granulation tissue and the rate of collagen deposition, which are known to expedite the healing process (Martins et al., 2003). In a similar study, the mucus of *Helix aspersa* demonstrated antimicrobial activity against several strains of *Pseudomonas aeruginosa* (Pitt et al., 2015). Further, the mucus of both *A. marginata* and *A. fulica*, were utilized as wound dressinsg on 28 clinical wound samples collected with known common infections (Etim et al., 2016). The mucus showed anti-bacterial potency against *Staphylococcus*, *Streptococcus,* and *Pseudomonas* isolated from wounds. In the same study, when compared to seven common antibiotics, including amoxicillin, streptomycin, and chloramphenicol, some of the mucus secretions were more inhibitory to infections than commercial antibiotics. Understanding the antimicrobial properties of snail mucus is an active and growing area of research.

**TABLE 1:** Mollusca species whose mucin have been applied in various sectors for biomedical or biotechnology applications.

Mollusca species	Common name	Applicable sectors	Uses	Development stage
*Helix aspersa* 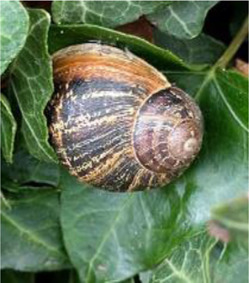	Garden Snail	Cosmetics	Skin Care Cancer Treatment Topical Antibiotic	Commercially available(Benton, Mizon, Cos Rx, Biopelle, Missha)
*Archachatina marginata* 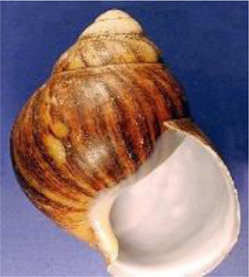	Banana Rasp Snail	Antimicrobial Pharmacology Wound Care	Antibiotic Drug Delivery & Medication Wound Dressing	Patented for use (US patent #: WO2000068258A2)
*Achatina fulica* 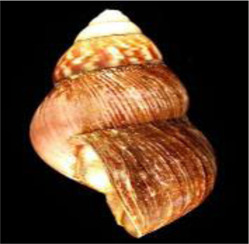	Kalutara snail	Antimicrobial Pharmacology Wound Care	Antibiotic Drug Delivery Medication Wound Dressing	Patented for use (US patent #: WO2000068258A2)
*Arion subfuscus* 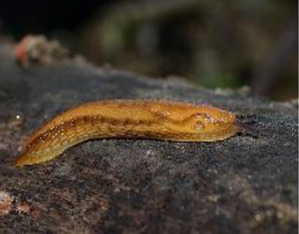	Dusky Arion	Medical equipment	Surgical glue	Active research (University of Pennsylvania Lehigh University)
*Helix pomatia* 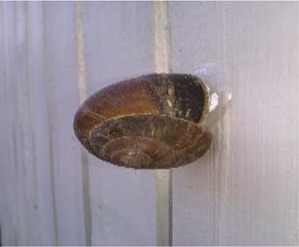	Burgundy snail	Personal care	Shampoo	Commercially available (Royer)
*Tikoconus costarricanus* 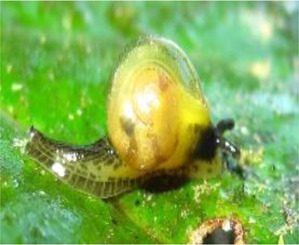	*T. costarricanus*	Biotech	Adhesion and lubrication	Reported in literature

**TABLE 2:** Molluscan mucin applied as antimicrobials to inhibit pathogens.

Mollusca species	Mucin tissue type	Dose	Bacteria	References
*Achatina fulica* 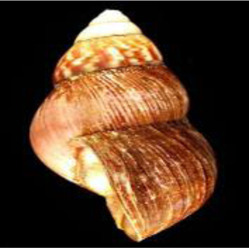	Foot	10 uL	*S. aureus*	Mumuni et al. (2020) Santana et al. (2012) Etim et al. (2016)
		10 uL	*S. epidermidis*	
		350 Ug/cm3	*B. subtilis*	
		350 Ug/cm3	*E. coli*	
		350 Ug/cm3	*P. aeruginosa*	
		4 mg/ml	*S. aureus*	
		4 mg/ml	*S. pyogenes*	
		4 mg/ml	*P. aeruginosa*	
*Helix aspersa* 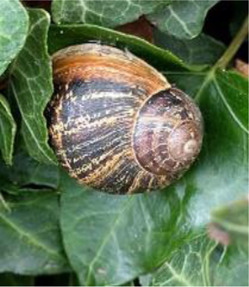	Foot	4.8 mg/ml	*P. aeruginosa*	Pitt et al. (2015)
*Archachatina marginata* 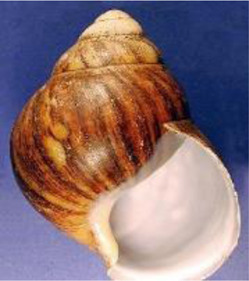	Foot	40% mucin/water mixture	*S. aureus*	Etim et al. (2016)
			*S. pyogenes*	
			*P. aeruginosa*	

### Snail Mucins as Drug Delivery Vehicles

The adaptability of snail mucin biopolymers makes them uniquely promising candidates for novel drug delivery systems (Huckaby and Lai, 2018; Momoh et al., 2020). During mating, male snails shoot a dart to deliver mucus containing accessory proteins into the female, which in turn increases the fertility of the female snail (Lodi et al., 2017). This process relies on a multifunctional system, with each component playing a defined role. The dart acts as a needle, piercing tissue and injecting the mucus that carries the accessory proteins into the female snail. In a similar manner, mucus could be adapted to act as a delivery vector for bioactive molecules. Snail mucus are known to pair exceptionally well with any medication that is absorbed via mucosal membranes because of their ability to facilitate diffusion across membranes (Balabushevich et al., 2019). For example, metformin hydrochloride, a diabetes medication, was attached to Giant African Land Snail mucin using polyethylene glycol (PEG) to increase bioavailability of the drug (Momoh et al., 2014). PEGylation, is a commonly utilized process where a therapeutic is surrounded by a matrix of polymers predominately, polyethylene glycol. This matrix advantageously affects the pharmacokinetics of the therapeutic, extending the half-life, via protection from antibodies, and allowing for variable control of biodistribution depending on matrix composition. Snail mucin in particular shows promise as a polymer, as they are highly hydrophilic, and also readily interact with gastrointestinal mucosal membranes, a common drug absorption location. Metformin-loaded PEGylated-mucin improved pharmacokinetics and pharmacodynamics of the normally poorly absorbed drug, increasing release to 92% compared to the 81% currently used in market. In another application, whole *Costus afer* (ginger lily) flowers combined with snail mucus showed reduction of blood glucose levels in diabetic *Mus musculus* (Swiss albino mice) in a dose-dependent fashion, which showed the possible anti-diabetic potential of snail mucin (Agu et al., 2018).

Drug-binding polymer matrix and mucin-containing vaterite crystals have been used as drug delivery carriers for effective loading and controlled release of small anti-cancer drugs and protein-based therapeutics (Balabushevich et al., 2019). Vaterite microcrystals, when crystallized in mucin concentrations ranging between 1 and 6 mg/ml, have better retention of cationic bioactivities and stability in physiological conditions Additionally, mucins have been coupled with photosensitizers in order to enhance targeting and optimize control of delivery into cancerous cells (Dutta et al., 2019). Self-assembled mucin multilayer capsules and mucin-containing microparticles are of particular interest for future studies of controlled release drug delivery mechanisms, particularly to overcome challenges of biocompatibility, biodegradability, and mucoadhesion (Balabushevich et al., 2018).

### Snail Mucins as Anti-Tumor Agents

Snail mucin has shown therapeutic potential against melanoma, one of the most dangerous skin cancers (Ellijimi et al., 2018). While new developments in cancer therapy have resulted in greater remission rates and longer life expectancies for those afflicted, these developments have not shown similar yields for melanoma (Rutkowski and Kozak, 2017). As treatment resistance is common for this cancer, there is an urgent need to find effective novel approaches for treating melanoma. A study of *H. apersa* mucus on melanoma cell lines reported that snail mucous decreased the viability and inhibited the metastasis of melanoma cells (Ellijimi et al., 2018). The decrease in viability of the cells was attributed to an apoptotic event related to cleavage of the Poly (ADP-ribose) polymerase (PARP). Additionally the inhibition of metastasis was achieved by blocking integrin function and expression, and thus inhibiting the cancer from being able to grow (Premi, 2020). In another study, *H. aspersa* mucin directly inhibited the growth of two human melanoma cell lines, by increasing expression of the cytokine TNFα, and inhibiting NF-κB, a transcription process that in proper regulation has been linked to cancer progression, the growth of these cell lines was decreased, demonstrating its anti-melanogenic properties (Domínguez-Martín et al., 2020). While still in the early stages of development, the application of snail mucins as anti-tumor agents is of growing interest in the biomedical community.

### Snail Mucins Facilitate Wound Healing

Snail mucus can facilitate healing and has become an important resource in wound research (Michael, 2012; Etim et al., 2016). Mucins from the *Helix aspersa* (Garden Snail) have been shown to help with skin regeneration after acute radiodermatitis, a common side effect from radiotherapy (Hymes et al., 2006). Garden snail mucus reportedly increased healing rates through antioxidant and free radical regulation (Nguyen et al., 2020). Mucus from garden improved erythema in rat models, and, the same rats showed reduction of photoaging as well (Lim et al., 2020). As well as being able to treat superficial injuries, mucins have shown the ability to be used on internal wounds. Mucins have been incorporated alongside oral non-steroidal anti-inflammatory drugs (NSAIDs), to reduce or eliminate gastric mucosal injury (Abdulla et al., 2013). NSAIDs reduce inflammation, but have adverse side effects related to gastrointestinal injury and liver damage. Many companies have turned to natural products to counteract these side effect, and Mucin have been shown to treat peptic ulcers, a side effects caused by NSAIDs (Drina, 2017). A combination of the antibiotic, clarithromycin, and *A. fulica* mucin has shown positive results in treatment of peptic ulcer disease (Mu et al., 2008; Kabakambira et al., 2018). In addition to anti-ulcer properties, the healing rate of ulcers increased with concentration of mucin and was faster than clarithromycin alone.

### Snail Mucus Used for Bioinspired Materials

Studying naturally occurring substances as a platform to build new materials has resulted in multiple revolutionary products, such as Lipitor, Penicillin, and Morphine. Similarly, mucins have been used as a biomaterial coating in order to reduce rejection of inorganic implants. Rejection of surgical implants due to infection results in over 1 million medical cases per year with the cost of the original surgery only being a fraction of the cost of treating the corresponding infection (Darouiche, 2004). Applying mucin-based films to polyethylene terephthalate, a common material used in medical implants, greatly reduced the immune response triggered by IgG and IgM absorption into the plastic (Sandberg et al., 2009; Galo Silva et al., 2019). The same study also showed that it reduced the activation of fibrinogen, a known inflammatory agent, when contacting the mucin coating as compared to the uncoated plastic. Mucins have been shown in other studies to reduce microbe reproduction on implanted devices (Co et al., 2015). Mucin-based technologies show immense promise for advancements in the field of biomaterials.

An example of mucins being used as biomaterials is the application of mucins in the synthesis of water-soluble hydrocarbons. By ligating mucin and/or mucin-mimicking compounds with a hydrophobic lipid chain, the hydrocarbon complex remained suspended in aqueous conditions, even after several months, while the non-complexed hydrocarbon would precipitate rapidly from solution (Chen et al., 2004). In another related study conducted by Combaa’s group, this property was applied to enhance glucose detection by creating a stabilized suspension of carbon nanotube-mucin complex for a sandwich-type glucose biosensor. The resulting bioanalytical device is 20% more sensitive and 40% faster than conventional devices that do not include this sensor design matrix (Comba et al., 2018).

Mucins, which come into contact with medications absorbed through mucosal membranes, can also be used in chromatography to assist in determining bioavailability and absorption through the membrane (Gargano et al., 2014). Porcine gastric mucin, bound to the silica column via amino propyl linkers, allowed for separation of drug molecules by the drug’s mucus membrane binding affinity. In another study mucin was anchored to a column using an ion-exchange with calcium-alginate, the mucin is immobilized, mimicking biological mucus membranes. Longer retention time of the molecule within the mucin column indicated high drug-mucin interaction, which is correlated to delayed bioavailability *in vivo* (Bhat, 1995). This adds another dimension to evaluate medications used in specific diseases that affect mucin production, such as cystic fibrosis (Abdullah et al., 2018).

The same porcine gastric mucin column has been used to evaluate flavor retention by the food industry. The mucin column was shown to mimic a bovine tongue for flavor retention, which reduces the need for and could potentially eliminate animal testing (Dinu et al., 2019). Mucins have been extensively studied in their role with flavor perception (Çelebioğlu et al., 2020). The presence of mucins within the oral cavity has been directly correlated to increased sedimentation of flavor-producing compounds, which in turn increases flavor perception (Dinu et al., 2020). This phenomenon is also being examinned as the cause for the loss of taste in old age (Pushpass et al., 2019a). Decreased levels of MUC7 in saliva has been noted in older individuals decreased taste (Pushpass et al., 2019b). This downregulation is believed to reduce mucoadhesion of the flavor molecules, leading to attenuated taste perception.

### A Snail’s Pace to Characterization of Mucin Molecules

Despite growing interest in the field, there are still many obstacles that prevent advancements in snail mucin research. Many snail species that have the potential for novel mucin discovery are often inaccessible because of their habitat. The lack of readily accessible biological material samples and difficulty in identifying mucin structures prevents the reliable synthesis of mucins in quantities sufficient for repeated experimentation. Several groups are investigating sustainable, scalable approaches to producing synthetic mucins, however the field is in its infancy (Petrou and Crouzier, 2018) While mucins that have been isolated from the *A. fulica* have been extensively studied, other species remain neglected (Park, 2011).

The most viable method for commercial mucin production remains extraction and isolation from animals, which does not allow for substantial yields for application without abundant animal capital and generally involves invasive methods. The complexity, abundance, and localization of glycosylation patterns on each mucin, in addition to various mucin glycoforms cause difficulty in employing common separation methods to purify, synthesize, and analyze mucin samples (Navarro et al., 2018). Mucins often undergo posttranslational modifications, such as O-sulfation, N-sulfation, and N-deacetylation that differentiate function between proteins (Krasnova and Wong, 2016). These posttranslational glycan modifications are a hurdle to mucin sample purification, characterization, and synthesis. A promising synthetic approach involves using recombinant bacteria, glycosyltransferase(GT)-mediated polymerization, and trans-glycosylation (Krasnova and Wong, 2016). However, these methods are insufficient to achieve industrially practical yields and will fail to generate the exhaustive set of glycoforms that comprise natural mucus gels. There is still difficulty in creating the O*-*glycosylation in yeast, and there are challenges involved in transferring glycosylation branches to chosen protein residues. These issues present a need for developing viable and high yield methods for synthesizing mucins using scalable chemistries, which would be the first step in using mucins as targeted therapies or treatments (Kwan et al., 2020).

Recent years have seen the emergence of -omic technologies (genomics, transcriptomics, proteomics, glycomics) that require minimal amounts of samples, allowing for the characterization of rare or poorly accessible snail samples (Gorson et al., 2015). A similar strategy to what has been done with snail venoms using venomics (Holford et al., 2018; Anand et al., 2019; Fassio et al., 2019), which pairs transcriptomic and proteomic methods with *de novo* sequence bioinformatic assembly programs to identify the genetic structure of snail venom putative peptide toxins, can be applied to characterize mucin genes and mucus proteins (Figure 1). Specifically, by taking the nucleotide sequences of assembled exomes, and then pairing that with proteomic mass values, we can confirm linear mucin protein structures. In this approach we extract mRNA from mucus glands or whole animal and through a bioinformatic pipeline, identify mucin genes and primary mucin protein sequences. A new initiative, the Comparative Animal Mucomics Project (CAMP) will apply a systematic comparative analyses of mucin genes and mucus hydrogels to determine the hierarchical structures and properties of distinct mucuses (Cerullo, 2020).

Despite the promise of omic methods for producing robust databases of mucins, major hurdles still remain for their study. One such hurdle are the algorithms used to assemble sequenced genes. De Bruijn graphs, which is the algorithm sequence most assemblers use, have difficulty mapping the repeated domains due to the multiplicity of similar overlapped sequences (Mikheenko et al., 2020). Multiple tools are currently being developed to overcome this problem (Jain et al., 2020). Each program changes the weighting of the k-mers that are used to construct the De Bruijn graphs in order to accommodate for the tandem repeats. For mucin proteomic study, the intermolecular interactions of mucins with other mucins causes an additional degree of difficulty. Mucins naturally will create multimers of themselves, connecting multiple proteins together in order to form a larger structure, which is regularly observed in nature (Javitt et al., 2019). In order to then obtain a single protein, the cojoined bonds must be broken, without also breaking the bonds of the single protein. However, mucin multimer bonds are difficult to reduce without also having an effect on the rest of a single mucin chain’s secondary structure. A trial and error procedure is currently used in mucin proteomic studies to generate single protein masses. New characterization and synthesis techniques will need to be established to accurately identify and fabricate snail mucins, and with an omics approach we may be able to determine the genotype to phenotype mapping necessary to understand and decipher the functionality differences found in each mucin sample.

## Concluding Remarks and Future Perspectives

Snails are found in nearly every biome, and environmental conditions appear to drive the diversity of mucin genes and versatility of mucus functions (Lang et al., 2007; Lang et al., 2016). Snail mucins have demonstrated biomedical and biotechnology potential, and are a bioinspired resource of significant promise (Figure 1). Characterization of snail mucins are limited not by their inherent value, but by access and the complexity of the molecule’s identification, purification and investigation. There are still several questions left to be answered about the properties of mucins and mucuses in relation to the applicable uses. This prospective demonstrates the high yield potential of snail mucins, and by utilizing an adaptable comparative omics pipeline, we can better understand these unique proteins, and their advantageous biological and chemical properties.
